# From Materia Medica to the Pharmacopoeia: Challenges of Writing the History of Drugs in India

**DOI:** 10.1111/hic3.12304

**Published:** 2016-04-13

**Authors:** Nandini Bhattacharya

**Affiliations:** ^1^ University of Dundee

## Abstract

Historians of indigenous medicine in colonial India have looked more closely at the changes, reinventions and reformulations of institutions of Ayurveda and Unani than at the cognitive content of the drugs themselves. The few historians who have examined the changing content of indigenous medicines have conceptualised the creation of materia medica of Indian drugs through two tropes: one of circulation (of specific drugs) through epistemological and geographic boundaries and the second, of marginalisation of certain other drugs either through a lack of textual legitimacy or the lack of the newly discovered ‘active principles’ within each drug. While these approaches have been useful, there is a case to be made for understanding the creation of formularies of Indian drugs in 19th and 20th centuries through the prism of medical praxis in India.

This essay engages with the problem of providing a historical narrative of drugs and therapeutic commodities in colonial India. This involves locating the histories of drugs within the conventional divides of ‘western’ and ‘indigenous’ medicine in colonial India. In the process, it will analyse the meanings of the multiplicity of drugs in the Indian market, the inclusions and exclusions of drugs from the formal and informal formularies, and explore the principal themes of circulation, marginalisation and formalisation of drugs in both text and praxis in colonial India. All of these were involved in the journey from numerous *materia medica* to the Indian Pharmacopoeia. An official Indian Pharmacopoeia was first published in 1955. There were, however, several *materia medica* and accounts of the ‘indigenous’ drugs of India from the 16th century onwards, and these became numerous in the 18th and 19th centuries. An exploration of the *materia medica* of colonial India, therefore, reveals not only the thousands of drugs, botanical, mineral and animal that were indigenous to India, but also reveals a history of the epistemological changes in both western and Indian therapeutics in this period.

Harkishan Singh, who has written extensively on the histories of the Indian pharmaceutical industry, pharmacy and pharmacopoeia in India, has tended to list the numerous pharmacopoeia in a chronological fashion and argued for the linear progression of Indian drugs in the British Pharmacopoeia (B.P.) from the time of the European discovery of indigenous drugs. This triumphant and seemingly uninterrupted journey, according to Singh, had culminated in the first official Indian Pharmacopoeia of 1955.^1^ This progression towards an Indian Pharmacopoeia, borrowing from and adding drugs of common use by the Indian practitioners, involved the gradual extension of the British pharmacopoeia to include some of these drugs. It was finally fulfilled with the compilation of an Indian Pharmacopoeia. The exclusions and marginalisation from the official formularies are of as much historical consequence as the inclusions of Indian drugs within the British Pharmacopoeia and the publication, finally, of an Indian Pharmacopoeia.

Apart from Singh's work, we do not have histories of the making of the Indian pharmacopoeia. In general, historians of indigenous medicine in South Asia have argued for the reformulation of the collective identities of the practitioners of Indian medical systems. These, they have argued, (with different regional variations) were facilitated by a vibrant vernacular and regional press, institutionalisation of their pedagogy through streamlining both texts and the practical lessons, reformulation of their packaging and use of innovative and modern marketing techniques – all of which were given an emotive edge by aligning Ayurveda and Unani with nationalist self‐assertion.^2^

The rich historiography of indigenous medicine, therefore, has succeeded in highlighting the problematics of modernising the pre‐modern histories of indigenous medicine. David Hardiman has pointed out that the revival of indigenous medical systems, both Ayurveda and Unani‐Tibb, involved reinventing (or to borrow Charles Leslie's term from his pioneering article), reviving Indian medical systems into new forms.^3^ This involved formulating both Ayurveda and Unani‐Tibb as cogent medical systems with a well‐defined structure. Hardiman has, following Romila Thapar's work on the Hindu syndication, suggested that both Unani‐Tibb and more tortuously, Ayurveda, were reformulated in colonial India: first, through Orientalist examinations from ancient and mediaeval scripts and second, through Unani and Ayurvedic practitioners themselves from the 1930s. These projects assumed both a corporate consensus and a ‘rational’ and ‘scientific’, Enlightenment method in order to first locate and then historicise their principal texts. Historicising the texts, of course, was difficult; not only were ‘ancient’ Sanskrit medical texts hard to obtain in the 19th century, but the few available were held within families who practised Ayurveda and were unwilling to disseminate that knowledge. Hardiman has argued that the reinvention of both Ayurveda and Unani as modern medical systems remained and continues to be patchy, because of lack of strong state support after independence as well as fragmented borrowings of biomedical therapies and technologies on the part of the indigenous medical practitioners.

While the historians above have provided us with deep histories of the reinvention of indigenous medicine in response to the challenges of the state‐patronised western medical institutions, they have paid curiously scant attention, with a few exceptions, to the materia medica – the content of the drugs themselves. Therefore, in the histories of indigenous medicine mentioned above, the cognitive content of the drugs themselves and their historic significance remain elusive.

There have been a few essays that historicize the journeys of drugs in colonial India. These tend to focus on their circulation across geographical and epistemic boundaries (within the indigenous medical paradigms), or refer to their marginalisation either in text or in practice. Generally, the erasures were the consequence of the chemical revolution in the 19th century that privileged the active principle within the drugs, relegating those drugs that were of multifarious origins, plural uses and diverse scientific characteristics to obscurity.

Meanwhile, the essays that analyse the histories of the drugs themselves have focussed on two principal themes: that of circulation of drugs and of the marginalisation of certain other drugs. Although these have been treated in two different themes, I believe that both the circulation and the marginalisation of specific drugs can be located within the problematic of the standardisation of drugs and in a broader sense, the legitimacy (or otherwise) of indigenous drugs.

## Circulation of Drugs Across Epistemic and Geographic Boundaries

Richard Grove's works include *Green Imperialism*, where he ingeniously argues for a botanical world‐view of French and British imperialists in the 19th century, as evidenced by their patronage of tropical gardens, conservation efforts (specifically in St Helena).^4^ Drawing on the same theme, Grove has argued in a separate essay for a genealogy of global trade in medicinal/botanical products and referred to the classification of drugs that occurred in the 17th century Goa.^5^ Garcia D'Orta's *Colloquis* and the Dutch botanist Van Reede's *Hortus Malabaricus* were written in the context of the Portuguese and later, the Dutch global trade. Interestingly, however, he has also focussed on the marginal position and perspectives, of Garcia D'Orta in particular, attributing these both to his hidden Jewish identity as well as his willingness to consider local, indigenous knowledge that was not dependent on textual Arabic (Unani) or even less on Ayurvedic practices. Grove's work has situated the first ethno‐botanies of the Indian sub‐continent in the context of a diffusion of indigenous botanical and medical knowledge into wider European medical and commercial networks. Grove's essay, however, does not go beyond a European perspective of indigenous drugs and their trade.

Guy Attewell's essay ‘Interweaving Substance Trajectories’ is an example of a more recent history of the circulation of drugs in the globalised world from the 18th to the 20th centuries.^6^ Through the prism of circulation, identification or misidentification Attewell untangles the complex wanderings of the tahriq‐al‐fareek or the Venetian Treacle across cultural and epistemic as well as social, temporal and geographical boundaries. In the process, he identifies the single drug/substance theriac/or tiryaq‐al‐faruk (Anglicised treeak faruk), the transmission of which encompassed pre‐colonial Mughal, Deccan, Ottoman and Venetian trading networks and made an irrelevancy, the author argues, of epistemological boundaries between Unani/indigenous or western medicine. The treeak faruk, imported from Venice to Masulipatnam through Arab traders in the 17th century, was used in British experiments with ‘native drugs’ in the 19th century Madras as a remedy for beri‐beri. It has survived in contemporary Unani therapeutics as a remedy for the H1N1 virus. The contents of the theriac undoubtedly changed over its travels, both temporal and spatial. So did its value; a luxurious commodity in the 18th century Hyderabad and used by princes as antidote to poisoning, it was a common ‘native’ remedy for rheumatism and oedema in the 19th century. Attewell argues for continuities: between trading networks of the 16th and the 19th centuries, as well as between epistemic incorporations in apparently discrete materia medica through the example of this drug.

Other historians have discussed a similar circulation of drugs over geographies and epistemological boundaries. Anna Winterbottom, for instance, has pointed out how the botanical drug known as the ‘China root’ used from the 16th century onwards for syphilis and related diseases was a valuable commodity that circulated across several trading networks in the early modern world, from China to Persia, and from Cairo to Venice and Padua.^7^ The China root, or the *Smilax China*, was traded from the Portuguese ports in the south‐east, including Macau and Malacca to the west coast of India; from here, they entered the Indian bazaar or country trade and were incorporated within Sanskrit and vernacular texts on Ayurvedic therapies and in Persian texts on Unani medicines in the 16th century. So the China root circulated not only in therapeutic texts but also in practice; it was extensively used in Safavid Persia and made entry into Mughal‐era therapeutic texts in the late 16th century. The British picked this up from their regular explorations within the Indian country trade in the 18th century, and at this time, it was used extensively to cure syphilis; no other western drug was found to be as efficacious.

In the process, the China root emerges as both a changing ‘thing’ or material of the 18th century global drugs trade as well as an illustration of a therapeutic product's journey to oblivion with its eventual substitution in the western pharmacopoeia with sarsaparilla in the 19th century. This involved, Winterbottom has argued, political exigencies as well as modern scientific universalism. Therefore, sarsaparilla, which had similar properties to the China root but could be cultivated large‐scale in the colonial West Indies, became a legitimate substitute for the China root, and finally, when its active component ‘Perillin’ could not be found in the ‘original’ China root, pharmacological science and political and economic opportunism both facilitated the expunge of the China root from the British pharmacopoeia by 1914.

Both Attewell and Winterbottom have privileged circulation of drugs within the early modern globalised world in their examination of the journeys of individual drugs. While Attewell leaves out the question of politics of circulation altogether, Winterbottom locates the movement of meanings and content of drugs within economic and cultural frameworks that made the difference between a drug's continuity in the pharmacopoeia, leading us beyond a simpler scientific model.

## Selective Marginalisation of Drugs and the Certitude of ‘Active Principles’

Other historians have argued for the circulation of drugs as a process of their marginalisation. In this, let us examine two other explorations of individual drugs: one within texts and the other in therapeutic praxis. The first is Projit Mukharji's essay on one ‘Vishalyakarani’, a mythological drug referred to in many readings of the epic *Ramayana.* 8 Mukharji draws upon the concept of ‘retro‐botanizing’ to deconstruct the historical narrative of the drug as an ancient Indian therapeutic; instead, it emerges as a Brazilian drug that had been imported to India only after the 15th century. Given contemporary political/nationalist discourse on the ancient nature of indigenous drugs, the *vishalyakarani* fell between the opposing categories of either ‘indigenous’ or ‘western’ in any meaningful sense. This incapacity to obtain an ancient and ‘Indian’, textual, Sanskrit lineage marginalised the drug in the newly reforming indigenous drugs' movements and their texts in colonial India. And to the extent, that indigenous drugs were visible in the British Pharmacopoeia, *vishalyakarani* was marginalised at that site as well. Its textual disappearance, nonetheless, did not obliterate its use in daily medical practice among Indian practitioners. Meanwhile, the quest for the ‘original’ *vishalyakarani* continues in contemporary Indian medical discourse.

This trope of marginalisation occurs too in Pratik Chakrabarti's investigation into the history of another specific medicinal plant, the *Swietenia febrifuga*.^9^ It emerged out of an imperial imperative to find viable and cheaper alternatives to the cinchona plant. The imperatives were commercial and scientific, because in the 18th century, most of the wild cinchona existed in South America which was outside the British imperium. Several alternatives to the cinchona were examined by scientists across the British Empire in the 18th century, including the *Cinchona officinalis* and the *Cinchona caribeana* as well as the *Cinchona triflora* in the British colonies of St Lucia and Jamaica. The *Swietenia febrifuga* was first discovered in Rajamundhry (present‐day Andhra Pradesh) in 1793 by W. Roxburgh, an EIC surgeon and botanist. He sent it to Britain for scientific assessment and recognition as a genuine substitute to cinchona. As Chakrabarti has pointed out, alternatives to cinchona were an intensely competitive field in imperial scientific circles. Chakrabarti argues that the isolation of the active principle, ‘cinchonine’ from the ‘original’ cinchona bark, relegated all other cinchona substitutes to an ‘analytic contraction’ in scientific discourse and medical praxis. This analytic contraction, he emphasises, was common to many colonial and peripheral drugs, scientific networks and researches; reduced to the clinical tests of the ‘active principle’ ingredients, many drugs were either marginalised or ignored. In fact, the *C. ledgeriana*, a variety of cinchona that yielded the greatest quality of cinchonine, was cultivated in Java and remained under the control of a Dutch cartel until the Second World War.^10^ Therefore, the analytic contraction and the legitimacy granted by the ‘active principle’ ingredient in each drug wrested drugs in the colonies from their social and cultural contexts and rendered many of them irrelevant in the 19th century. On a broader canvas, Chakrabarti has argued that the circulation of drugs within the globalised world of the 18th century can only be understood in terms of the enrichment of both intellectual and material wealth of European imperialism.^11^

The tropes of circulation and marginalisation have enriched our understanding of the complexities of the making of an Indian materia medica. The trope of ‘circulation’ divests the drugs from their political and cultural contexts and fails to note the marginalisation and erasure of certain drugs. At the same time, I would argue also that too strong an emphasis on marginalisations tend to obscure the nuances of the inclusions within the pharmacopoeia. These nuances are significant because they carry within them the tortuous routes from indigenous drugs to a more generic pharmacopoeia. An examination of these inclusions could as well relate a history of the power of the imperial knowledge to transform an indigenous drug to a metropolitan one. One example is that of the seeds and oil of chaulmugra which was used extensively by both Ayurveds and Hakims as a cure for leprosy. It was incorporated within the British Pharmacopoeia, and in 1909, the German manufacturer Bayer had even obtained a patent for it.^12^ Does the journey of chaulmugra from a bazaar drug to a patented therapeutic by Bayer (an inclusion) can as well tell us something about power relations within the global drugs market? Stuart Anderson, who in a brief article has explored the history of the British Pharmacopoeia (B.P.) in India, emphasises that between 1868 and 1932, the B.P. (and its Indian and colonial addendum, first published 1900) emerged as the standard of quality in medicines in India.^13^ Therefore, the B.P. did become, he concludes, an imperial ‘tool’ and in turn was a catalyst to the nationalist Indian demand for an Indian Pharmacopoeia. While Anderson is correct in assuming that the B.P. was held to be the standard of dispensing medicine in colonial India, his investigations have remained limited to the circulation of the B.P. and its special colonial addendum. A closer attention to the medical market in India might enable historians to rethink his assumption that in India, medicines were expected to be of British Pharmacopoeia standard. In the absence of drug adulteration legislation of the kind prevalent in Britain, all drug stores in India, including British‐owned ones, sold medicines that did not conform to the B.P. The B.P. was a hallmark of quality in name, but rarely available in fact.

Moreover, particularly between the two World Wars, research on the potency and characteristics of Indian drugs occurred at various Indian medical research institutions, not all of them motivated by cultural nationalism. Instead, the intense research and publications over the legitimacy of specific Indian drugs, transcending cultural epistemologies of Ayurveda/Unani/western therapeutics, seem rather to emphasise the richness and diversity of the Indian medical market and the desire among medical researchers to harness these to enable the cheaper, easier, more durable alternatives to imported medicines from Europe and elsewhere. Therefore, the question of praxis remains critical in understanding the history of the Indian Pharmacopoeia.

## Towards a History of the Indian Pharmacopoeia

In an attempt to resolve the challenge of writing the history of the Indian Pharmacopoeia, I would like to query if it is possible to understand this history in a different form than the linear, triumphalist history that Harkishan Singh provides.

The history of the Indian therapeutics has evolved along with the history of the Dutch and British discovery of centuries‐old Indian medical traditions. In that sense, Whitelaw Ainslie's *Materia Medica of Hindoostan* (1813) was the first collection that opened up the study of Indian botanical therapeutics to the English speaking world.^14^ Ainslie, who was a surgeon at the Madras medical establishment, wrote this as a useful catalogue ‘of such medicines of the British *Materia Medica*, as are either the produce of Hindoostan, or brought to it from Asiatic countries, and are to be met with in the Bazaars of populous Towns….’ This catalogue, an exhaustive list of the medicines available in the bazaars and used by Indian and British medical men, was an eclectic collection of botanical and mineral therapeutic products found in the local markets. And although stating the botanical and English names for many products, pragmatically allowed for the local names in Telugu, Tamil, Dakhini and Sanskrit for any drugs or minerals for those products that for which he could not identify botanical names. His catalogue, published in two large volumes of around 600 pages each, also identified British therapeutic products used by Indian physicians. In that sense, Ainslie was the first historian of the medical praxis as evident in the medical marketplace.

Now, Ainslie's work, with its textual and epistemological certitude, can also be read as the beginning of the textual marginalisation of indigenous drugs. And this marginalisation occurred also at the level of the dispensers of these drugs. Projit Mukharji has argued that ‘subaltern herb gatherers’ were marginalised by Indian drug traders and pharmacists at this time.^15^ Similarly, Rachel Berger has argued that Indian drugs in general were written out of pharmacopoeia by the late 19th century. However, as I have pointed out above, such an approach cannot explain the retention of the various bazaar medicines in practice.^16^

This retention is evident in John Waring's *Remarks on the Uses of Some of the Bazaar Medicines and Common Medical Plants of India* published in 1859.^17^ Waring's *Bazaar Medicines* proved enormously popular and had made four editions by 1883. The first edition had been bi‐lingual, in Tamil and English, and printed for issuing to local vaccinators in the Madras medical establishment. Local vaccinators evidently also functioned unofficially as both medical practitioners and medical informants on local medical materials until the late 19th century. By the third edition, Waring expected these formularies to be used widely in remote areas in India. Thus, it is necessary to read Waring's ‘bazaar’ as the site of the praxis of indigenous drugs, and praxis therefore is key to explaining the emergence of the Indian Pharmacopoeia.

Perhaps this trajectory of an Indian pharmacopoeia will be clearer with an analysis of Kanny Lall Dey's more modest compilation. Dey was a western educated physician and a much‐respected member of the first Indian Medical Congress. His *Indigenous Drugs of India: Short Descriptive Notices of the principal medicinal products met with in British India* was first published in 1867 and then revised and re‐written in 1896. This represented his life‐long aspiration to collect, categorise and, finally, to fully integrate as many Indian drugs with western *materia medica* as possible. In 1885, Dey was the only Indian nominated to the first of many committees by the Government of India to make a comprehensive list of Indian medicinal plants.^18^ In 1899, the *Chemist and Druggist*, a prominent trade journal published from Britain, dismissed the endeavour to integrate indigenous medicines within the British Pharmacopoeia: ‘so far, the committee has been little else than called into existence; it has met and corresponded, but does not feel clear as to what it is all about, or how it can benefit mankind by investigating things that are well known, so practically no useful end appears to have been served by the committee’.^19^

To return to the B.P., with its unprecedented colonial addendum, these were less celebrated than might be expected in India, particularly by British officials, who largely dismissed them as either academic or purely historical. An editorial in the *Indian Medical Gazette* even alleged that it was not undertaken in consultation with the Government's own committee on indigenous drugs. The addendum, in any case, did not impact to any greater extent on the use of substitutes of western formularies with Indian ones already in use. Therefore, the textual marginalisation had little effect on the use of the drugs within the medical market. Their marginalisation had already been effected by this time.

This is apparent from a study of William Dymock's compilation. After Ainslie's work, the next voluminous addition in English to the Indian *materia medica* was the William Dymock's *Pharmacographica Indica*.^20^ This was a four‐volume magnum opus, and a follow‐up of his *The Vegetable Materia Medica of Western India,* published a decade previously.^21^ Dymock's volumes were a huge, collaborative attempt to document the botany of the Indian subcontinent as comprehensively as possible. In this sense, they added to colonial epistemology in the same way as the decennial census, the trigonometrical surveys and maps of the Indian sub‐continent over the 19th century. Dymock was exceptional by the late 19th century. J. F. Royle, whose *Essay on the Antiquities of Hindoo Medicine* privileged ancient Indian medicine as historically preceding Greek and Arabic medicine (in an evolutionary paradigm) nonetheless confined his collection of ‘Hindu’ *materia medica* to materials within the London Pharmacopoeia (1851) and British Pharmacopoeia (1864).^22^ There was a practical reason: the Bengal Board had commissioned Royle to compile a list of drugs available in the Indian bazaars that might be conveniently used as substitutes for the much more expensive imported items. Royle's *Antiquities* listed around 1000 botanical and mineral items sold in Indian bazaars as country medicines. In his *Manual of Materia Medica and Therapeutics*, which went through several editions, Royle made it clear that his previous work was relevant to the extent that the histories of some drugs ‘investigated by Hindoos’ and used in the present could be identified.^23^

Yet the volumes of the *Pharmacographica Indica*, unlike that of Royle's compilation, reveals more interesting ways in which the materia medica of India was evolving systematically towards a framework of pharmacological knowledge that would eventually be divested from its social and cultural roots. Therefore, historiographically, Dymock poses a problem. Can we take Dymock's account of ‘mythological’ and ‘historical’ Indian drugs as proof of western engagement with Indian drugs, particularly in the context of their globalisation? Meanwhile, Chakrabarti has pointed out that the discovery of the ‘active principles’ in therapeutic commodities in the mid‐19th century (caffeine from coffee, morphine from opium and, particularly, quinine from cinchona) separated the drugs from their cultural origins and even more significantly, from the political contexts of their discovery, and collection, and the processes through which they were assimilated into western pharmacopoeia. No doubt this was a long and varying process; Dymock's *Pharmacographia Indica* claimed that the collection had put together scattered publications on Indian pharmacopoeia, often from obscure sources, as well as ‘endeavoured to collect and verify this information, and supplement it where deficient by original investigation especially…the chemical composition and physiological action of the plants and drugs’. So far, Dymock seems to have been in line with the general trajectory of dissociating drugs from their broader contexts into chemistry‐oriented formularies. British physicians and pharmacists, including surgeons appointed at the government medical hospitals, still used ‘bazaar’ or ‘country’ medicines when European drugs were not available when *Pharmacographia Indica* was published.

There is no doubt that Dymock's *Pharmacopeia Indica* was used regularly by pharmacists and drugs merchants in the 19th century and the early 20th century India. The trade journal in London, *Chemist and Druggist*, used Dymock's work to emphasise the substitution of belladonna with the indigenous *datura fastuosa*, a relatively new substitution.^24^ This, I suggest, was a different kind of substitution from the 18th century bazaar substitutes used when European drugs were not available – it involved a deeper ingraining of the Indian drugs market with the global demands, and this was accepted by British authors of pharmacopoeia. This was a new globalisation of Indian drugs. As Kanny Lall Dey suggested in 1884, Calcutta had the potential of being established as a ‘drug emporium which would be central to the great markets of the world....’^25^

But there is a disjuncture here. The preface to the *Pharmacographia Indica* itemises unexpected materials that are difficult to fit into the above mentioned trajectory. ‘Plants of historical and mythological origins’ write the authors, ‘are not omitted, as the history of Indian medicaments would be incomplete without them.’ This presents us with a difficulty: was Dymock's collection a pharmacopoeia or a history of *materia medica* in the Indian sub‐continent, and if it was both, does this subvert the linear account of the movement from materia medica to pharmacopoeia? Why in short, did Dymock devote so much time and energy to include ‘plants of historical and mythological origins’^26^? He confirmed that these ‘though possessing little or no medicinal activity, have not been omitted, as the history of Indian medicaments would be incomplete without them’. At the same time, Dymock articulated his wish to engage contemporary (presumably western) medical practitioners with his collection and provided comparisons of ‘empirical estimates’ of drugs with modern pharmacological research. These included a lengthy list of poisons as well as obscure drugs that were not used by Indians, such as the *Meconopsis Wallichii* (112) which did not have a vernacular Indian name; or the *Kaff Maryam*, imported from Syria, and whose efficacy the author appears to have had no opinion of. One possibility is that by enumerating the mythological or ‘historical’ drugs, Dymock sought to both demystify them as well as emphasise their mythological nature. As for ‘historical’ drugs, cataloguing them comprehensively served to highlight their contemporary irrelevance. In that sense, Dymock's work emerges as historical record as well as the guardian of contemporary formulary.

In conclusion, I would argue that the various pharmacopoeia have to be historically understood as the product of the drugs and therapeutics market in India, which was defined as a regime of praxis. A history of the numerous pharmacopoeia, therefore, needs to look at the structures of the drug market as well as the circulations and marginalisation in medical texts.

## Notes

1 Singh, *Pharmacopeias and Formularies*, 1994.

2 Sivaramakrishnan, *Old Potions, New Bottles* 2006; Alavi, ‘Unani Medicine in the Nineteenth‐century Public Sphere’, 2005,101‐129; Attewell, *Refiguring Unani Tibb,*2007; Berger, *Ayurveda Made Modern* 2013; Sharma*, Indigenous and Western Medicine* 2012*.*

3 Hardiman, ‘Indian Medical Indignity’, 2009: 263–283.

4 Grove, *Green Imperialism*, 1995.

5 Grove, ‘Indigenous Knowledge and the Significance of South‐West India’ 1996, 121–143.

6 Attewell, ‘Interweaving Substance Trajectories’, Digby, et al., *Crossing Colonial Historiographies*, 2010, 1–20.

7 Winterbottom, ‘Of the China Root’, 2014, 22–44.

8 Mukharji, ‘Vishalyakarani as Eupatorium Ayapana’, 2014, 65–87.

9 Chakrabarti, ‘Empire and Alternatives’, 2010, 75–94.

10 Bhattacharya, ‘The Logic of Location’, 2011, 183–202.

11 Chakrabarti, *Materials and Medicine*, 2010,

12 Nandini Bhattacharya, ‘Between the Bazaar and the Bench’, Spring 2016.

13 Anderson, ‘Pharmacy and Empire’ 2010, 112–121.

14 Ainslie, *Materia medica of Hindoostan*, 1813.

15 Mukharji, ‘Pharmacology, “Indigenous knowledge,” Nationalism’, in Pati and Harrison (eds.) *The Social History of Health and Medicine* 2009, 195–212.

16 Berger, ‘Ayurveda and the Making of the Urban Middle Class’ Wujastyk et al. (eds.), *Modern and global Ayurveda*, 2008, 101–113.

17 Waring, *Remarks on the uses of Some Bazaar* Medicines, Trivandrum, 1860.

18 ‘The Use of Indigenous Drugs in India’, *The Pharmaceutical Journal* 1895, p.464.

19 ‘Indigenous Drugs of India’, *Chemist and Druggist*, 1899, 536.

20 Dymock, *Pharmacographia Indica*, 3 vols. 1890–1893.

21 Dymock, *The Vegetable Materia Medica* 1884.

22 Royle, *An Essay on the Antiquity of Hindoo* medicine, 1847, vi.

23 Royle, *Manual of Materia Medical and Therapeutics*, 1847.

24
*The Chemist and Druggist*, 1888, 552.

25
*The Pharmaceutical Journal and Transactions*,1895 ,651.

26 Dymock, Pharmacographica *Indica*, Vol 1, Part 1, iii.
